# A Metabolism-Related Radiomics Signature for Predicting the Prognosis of Colorectal Cancer

**DOI:** 10.3389/fmolb.2020.613918

**Published:** 2021-01-07

**Authors:** Du Cai, Xin Duan, Wei Wang, Ze-Ping Huang, Qiqi Zhu, Min-Er Zhong, Min-Yi Lv, Cheng-Hang Li, Wei-Bin Kou, Xiao-Jian Wu, Feng Gao

**Affiliations:** ^1^Department of Colorectal Surgery, The Sixth Affiliated Hospital of Sun Yat-sen University, Guangzhou, China; ^2^Guangdong Provincial Key Laboratory of Colorectal and Pelvic Floor Diseases, Supported by National Key Clinical Discipline, Guangdong Institute of Gastroenterology, Guangzhou, China; ^3^Department of Gynecology, Huzhou Maternity & Child Health Care Hospital, Huzhou, China

**Keywords:** radiomics, colorectal cancer, prognosis, nomogram, metabolism

## Abstract

**Background:** Radiomics refers to the extraction of a large amount of image information from medical images, which can provide decision support for clinicians. In this study, we developed and validated a radiomics-based nomogram to predict the prognosis of colorectal cancer (CRC).

**Methods:** A total of 381 patients with colorectal cancer (primary cohort: *n* = 242; validation cohort: *n* = 139) were enrolled and radiomic features were extracted from the vein phase of preoperative computed tomography (CT). The radiomics score was generated by using the least absolute shrinkage and selection operator algorithm (LASSO). A nomogram was constructed by combining the radiomics score with clinicopathological risk factors for predicting the prognosis of CRC patients. The performance of the nomogram was evaluated by the calibration curve, receiver operating characteristic (ROC) curve and C-index statistics. Functional analysis and correlation analysis were used to explore the underlying association between radiomic feature and the gene-expression patterns.

**Results:** Five radiomic features were selected to calculate the radiomics score by using the LASSO regression model. The Kaplan-Meier analysis showed that radiomics score was significantly associated with disease-free survival (DFS) [primary cohort: hazard ratio (HR): 5.65, 95% CI: 2.26–14.13, *P* < 0.001; validation cohort: HR: 8.49, 95% CI: 2.05–35.17, *P* < 0.001]. Multivariable analysis confirmed the independent prognostic value of radiomics score (primary cohort: HR: 5.35, 95% CI: 2.14–13.39, *P* < 0.001; validation cohort: HR: 5.19, 95% CI: 1.22–22.00, *P* = 0.026). We incorporated radiomics signature with the TNM stage to build a nomogram, which performed better than TNM stage alone. The C-index of the nomogram achieved 0.74 (0.69–0.80) in the primary cohort and 0.82 (0.77–0.87) in the validation cohort. Functional analysis and correlation analysis found that the radiomic signatures were mainly associated with metabolism related pathways.

**Conclusions:** The radiomics score derived from the preoperative CT image was an independent prognostic factor and could be a complement to the current staging strategies of colorectal cancer.

## Introduction

Colorectal cancer (CRC) is one of the most common cancers and ranks as the third cause of cancer-related mortality worldwide (Siegel et al., 2020). Even with the recent progress in cancer treatment, the 5 years overall survival of CRC remains <60% (Moghimi-Dehkordi and Safaee, 2012). Traditionally, the treatment regime of colorectal cancer was mainly determined according to clinicopathological factors, such as the TNM stage, tumor size, differentiated grade, which didn't fully consider the heterogeneity of tumors. The emergence of gene expression-based molecular biomarkers has brought hope for the precision treatment of colorectal cancer in the past decade, but the high cost and long detection time limited its clinical application. In recent years, the medical images, which were routinely detected in clinical practice, have emerged to be promising biomarkers for cancer treatment and management.

Radiomic is a multidisciplinary approach concerning the quantification of medical images, like CT and magnetic resonance imaging (MRI). By transforming medical images into high-dimensional quantitative feature data, radiomics have been successfully used in some medical researches, such as tumor genetic analysis, lesions qualitative, curative effect evaluation and prognosis prediction (Kumar et al., 2012; Lambin et al., 2017; Limkin et al., 2017). Typical radiomic features describe the tissue or lesion characteristics, such as tumor shape, tumor texture, which can provide abundant information for tumor assessment. Compared with traditional clinical diagnosis methods, radiomics has the advantages of cheap, non-invasive, and quantifiable.

Several studies have demonstrated that radiomics analysis combined with clinicopathological information can largely contribute to guiding treatment decisions. Huang Y. Q. et al. (2016) developed a radiomic nomogram incorporating clinical risk factors for preoperative prediction of lymph node metastasis in patients with colorectal cancer. Similarly, CT-based radiomics signature in colorectal cancer shows considerable potential discrimination in preoperative staging (Liang et al., 2016). Kim et al. (2015) reported that some distinct features extracted from CT images can significantly discriminate differentiated grades on colorectal adenocarcinoma. In terms of prognostic evaluation, radiomic features are regarded as independent biomarkers for assessing disease-free survival in patients with early NSCLC. By combining with traditional staging systems and other clinicopathological risk factors, the radiomics signature achieved more effective performance (Huang Y. et al., 2016). Farhidzadeh et al. (2016) found that the radiomic features extracted from MRI images in patients with nasopharyngeal carcinoma (NPC) embody the heterogeneity of the tumor, showing high recurrence prediction in two groups patients. In addition, it has been reported that radiomic features based on CT images may correlate with genomics data underlying clinical outcomes (Segal et al., 2007; Aerts et al., 2014). Therefore, analyzing image radiomic features and excavating the hidden biological information have become a promising direction of image biomarkers research.

In this study, we aimed to develop and validate a radiomics-based nomogram to predict the postoperative outcome of colorectal cancer patients. RNA-seq data from the colorectal cancer subproject (COCC, Clinical Omics Study of Colorectal Cancer in China) of the ICGC-ARGO project (The International Cancer Genome Consortium-Accelerating Research in Genomic Oncology) were used to explore the underlying biological interpretation of the radiomic signature.

## Materials and Methods

### Data Collection

A total of 381 patients with colorectal cancer from The Sixth Affiliated Hospital of Sun Yat-sen University were enrolled in this study. Our study was approved by the Medical Ethics Committee of the Sixth Affiliated Hospital of Sun Yat-sen University. Patients admitted during 2007–2011 were assigned to the primary cohort (*n* = 242), while patients admitted during 2012–2015 were assigned to the validation cohort (*n* = 139). Fifty three patients of 381 patients were enrolled in the COCC project, so they have paired image data and RNA sequencing data. All the CT images are DICOM (Digital Imaging and Communications in Medicine) format from the image archiving and storage system of the Six Affiliated Hospital of Sun Yat-sen University. Baseline clinicopathological information containing age, gender, differentiated grade, lymph node metastasis and carcinoembryonic antigen (CEA, normal <5 ng/ml, abnormal > 5 ng/ml) were also derived from the hospital archives. Region of interest (ROI) was manually delineated on the tumor outline by skilled doctors using the ITK-snap (Version 3.2). A total of 107 radiomic features were generated using pyradiomics (van Griethuysen et al., 2017) package in python 2.7 platform.

### Model Construction

Z-score normalization for radiomic features was used to increase comparability. Only features with high intensity were retained for the following analyses. The least absolute shrinkage and selection operator (LASSO) with cox regression was used to construct the radiomic signature and calculate the radiomics score (Rad-score). A nomogram was constructed by incorporating the radiomics score with clinicopathological risk factors. The performance of the nomogram was evaluated by the calibration curve, receiver operating characteristic (ROC) curve and C-index statistics.

### Correlating the Radiomic Features With Gene Expression Data

To explore the association between the radiomic features and the underlying biological mechanism, we conducted a correlation analysis between radiomic features and cancer-related hallmarks. DeepCC (Gao F. et al., 2019) was used to calculate the enrichment score of hallmarks of cancer for each patient. The Pearson's correlation coefficient between each hallmark and radiomic feature was calculated. Hallmarks that have a significant correlation with at least one radiomic feature were displayed in the heatmap.

### Statistical Analysis

All statistical analyses were performed by R software (version 3.6.1). Time-dependent ROC curve was used to determine the optimal cut-off value of the radiomics score by “survivalROC” (Heagerty et al., 2000), which can divide patients into different risk groups. The R package “glmnet” was used to perform the LASSO-cox regression analysis (Friedman et al., 2010). Kaplan-Meier curves and log-rank tests were used to perform survival analysis. The primary outcome is disease-free survival (DFS). Univariable and multivariable analyses were performed by the cox proportional hazards regression model. Nomogram incorporating Rad-score with clinicopathologic factors was built by the “rms” packages (Harrell, 2016). The two-sided value of *P* < 0.05 was considered statistical significance in all analyses.

## Result

### Features Selection and Model Construction

**Figure 1:**
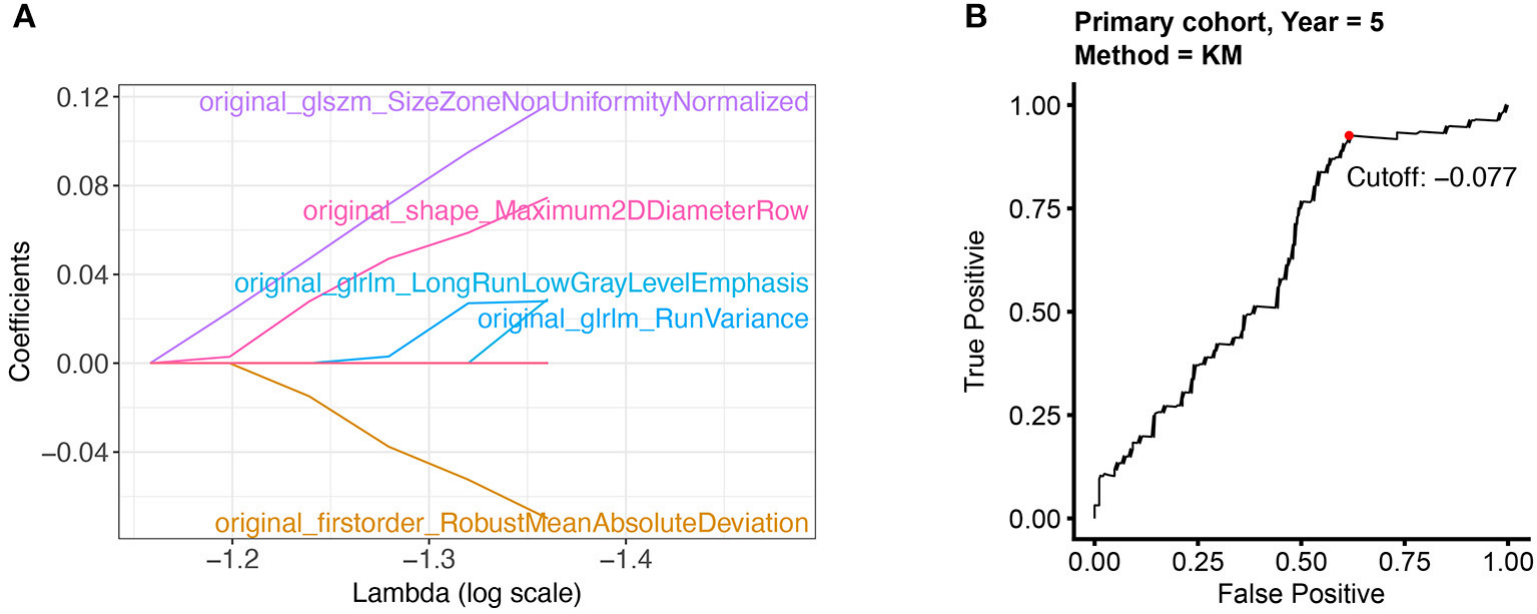
Selecting the radiomic features and the optimal cut-off point. **(A)** 5 radiomic features with non-zero coefficients were selected by using the LASSO algorithm. **(B)** The optimal cut-off point was determined by the time-dependent ROC curve.

In the preprocessing step, radiomic features were first scaled with z-score normalization in the primary and validation cohort. The average signal values of each feature in different patients were calculated and compared. We only retained 85 features (80% of 107 features) with higher signal intensity for subsequent modeling. Further, LASSO-cox regression was applied to select 5 features with non-zero coefficients (Figure 1A). Radiomics score was calculated by a linear combination of non-zero coefficients, which was multiplied by the 5 features vectors in the primary and validation cohort, respectively. The radiomics scores of all patients were displayed in Supplementary Table 1. The calculation process was presented in the following formula:

$$\begin{array}{l} Rad-score=\text{original\_shape\_Maximum}2\text{DDiameterRow}\times 0.075\\ +\text{original\_firstorder\_RobustMeanAbsoluteDeviation }\times \;\left(-0.070\right)\\ +\text{original\_glrlm\_LongRunLowGrayLevelEmphasis}\times 0.029\\ +\text{original\_glrlm\_RunVariance}\times 0.028\\ +\text{original\_glszm\_SizeZoneNonUniformityNormalized}\times 0.116 \end{array}$$

### High Radiomics Score Was Associated With Poor Outcome in Colorectal Cancer Patients

**Figure 2:**
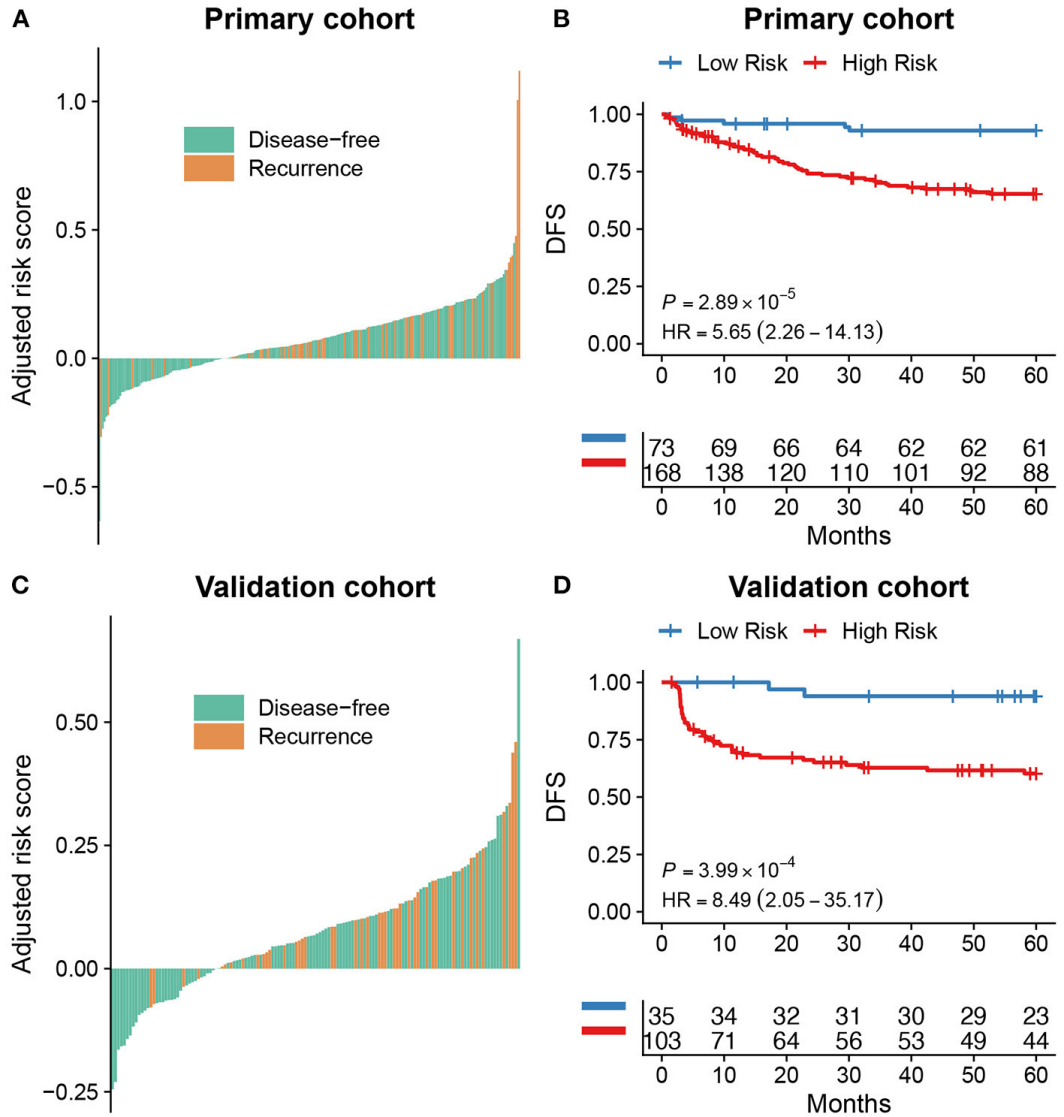
Survival analysis for radiomics score. The distribution of radiomics score in colorectal cancer and its correlation with recurrence status in the primary **(A)** and validation cohort **(C)**. Kaplan-Meier curves showed a significant association between Rad-score groups and disease-free survival (DFS) in the primary cohort **(B)** and validation cohort **(D)**.

The optimal cut-off of Rad-score was determined by the time-dependent ROC curve. Based on the threshold, the patients were divided into the high-risk (>-0.077) and low-risk (< -0.077) groups (Figure 1B). Patients' clinical characteristics in the primary and validation cohort were presented in Table 1. Survival analysis revealed a significant association between radiomics score and DFS in the primary cohort (HR: 5.65, 95% CI: 2.26–14.13, *P* < 0.001) and validation cohort (HR: 8.49, 95% CI: 2.05–35.17, *P* < 0.001) (Figure 2). Patients with a high Rad-score showed a significantly poor outcome. To adjust for the confounding effect of clinicopathological factors, the Rad-score, sex, age, carcinoembryonic antigen (CEA) level, differentiation grade, and TNM stage were added into the multivariable analysis (Table 2). Multivariable analysis demonstrated that Rad-score was an independent prognostic predictor of recurrence in the primary cohort (HR: 5.35, 95% CI: 2.14–13.39, *P* < 0.001) and validation cohort (HR: 5.19, 95% CI: 1.22–22.00, *P* = 0.026).

**Table 1:** Baseline characteristic of patients in the primary and validation cohort.

		**Primary cohort (*****n*** **=** **242)**			**Validation cohort (*****n*** **=** **139)**		
	**Level**	**Low risk**	**High risk**	***P***	**Low risk**	**High risk**	***P***
*n*		73	169		35	104	
Patients with survival data (*n*)		73	168		35	103	
Sex (%)	F	46 (63.0)	61 (36.1)	<0.001	19 (54.3)	40 (38.5)	0.15
	M	27 (37.0)	108 (63.9)		16 (45.7)	64 (61.5)	
Age [mean (SD)]		71.6 (13.2)	68.8 (14.8)	0.16	65.3 (14.8)	64.0 (12.6)	0.63
Differentiation grade (%)	High	18 (25.0)	45 (31.2)	0.15	10 (28.6)	27 (27.3)	0.54
	Moderate	50 (69.4)	82 (56.9)		19 (54.3)	46 (46.5)	
	Low	4 (5.6)	17 (11.8)		6 (17.1)	26 (26.3)	
TNM stage (%)	I	9 (12.3)	8 (4.7)	0.02	9 (25.7)	7 (6.9)	<0.01
	II	23 (31.5)	68 (40.2)		14 (40.0)	33 (32.4)	
	III	32 (43.8)	54 (32.0)		10 (28.6)	34 (33.3)	
	IV	9 (12.3)	39 (23.1)		2 (5.7)	28 (27.5)	
CEA (%)	Low (<5 ng/ml)	52 (71.2)	110 (65.1)	0.43	27 (79.4)	59 (57.3)	0.04
	High (>5 ng/ml)	21 (28.8)	59 (34.9)		7 (20.6)	44 (42.7)	
Lymph node metastasis (%)	No	34 (46.6)	82 (48.5)	0.89	23 (65.7)	48 (46.2)	0.07
	Yes	39 (53.4)	87 (51.5)		12 (34.3)	56 (53.8)	

**Table 2:** Univariable and multivariable analysis of clinical factors in the primary and validation cohort.

	**Primary cohort**				**Validation cohort**			
	**Univariable analysis**		**Multivariable analysis**		**Univariable analysis**		**Multivariable analysis**	
	**HR (95% CI)**	***P***	**HR (95% CI)**	***P***	**HR (95% CI)**	***P***	**HR (95% CI)**	***P***
Rad-score	5.65 (2.26–14.13)	<0.001	5.35 (2.14–13.39)	<0.001	8.49 (2.05–35.17)	<0.001	5.19 (1.22–22.00)	0.03
Sex	1.26 (0.74–2.12)	0.39			1.41 (0.75–2.67)	0.28		
Age	1.00 (0.98–1.02)	0.76			0.99 (0.96–1.01)	0.27		
CEA	2.01 (1.20–3.36)	<0.01	1.25 (0.72–2.16)	0.43	2.55 (1.37–4.76)	<0.01	0.96 (0.49–1.86)	0.90
Differentiated grade	1.12 (0.69–1.79)	0.65			1.87 (1.20–2.92)	<0.01	1.80 (1.12–2.89)	0.02
TNM stage	2.51 (1.79–3.52)	<0.001	2.30 (1.62–3.28)	<0.001	4.82 (3.04–7.63)	<0.001	4.82 (2.86–8.13)	<0.001

### Construction and Performance of the Radiomics Nomogram

**Figure 3:**
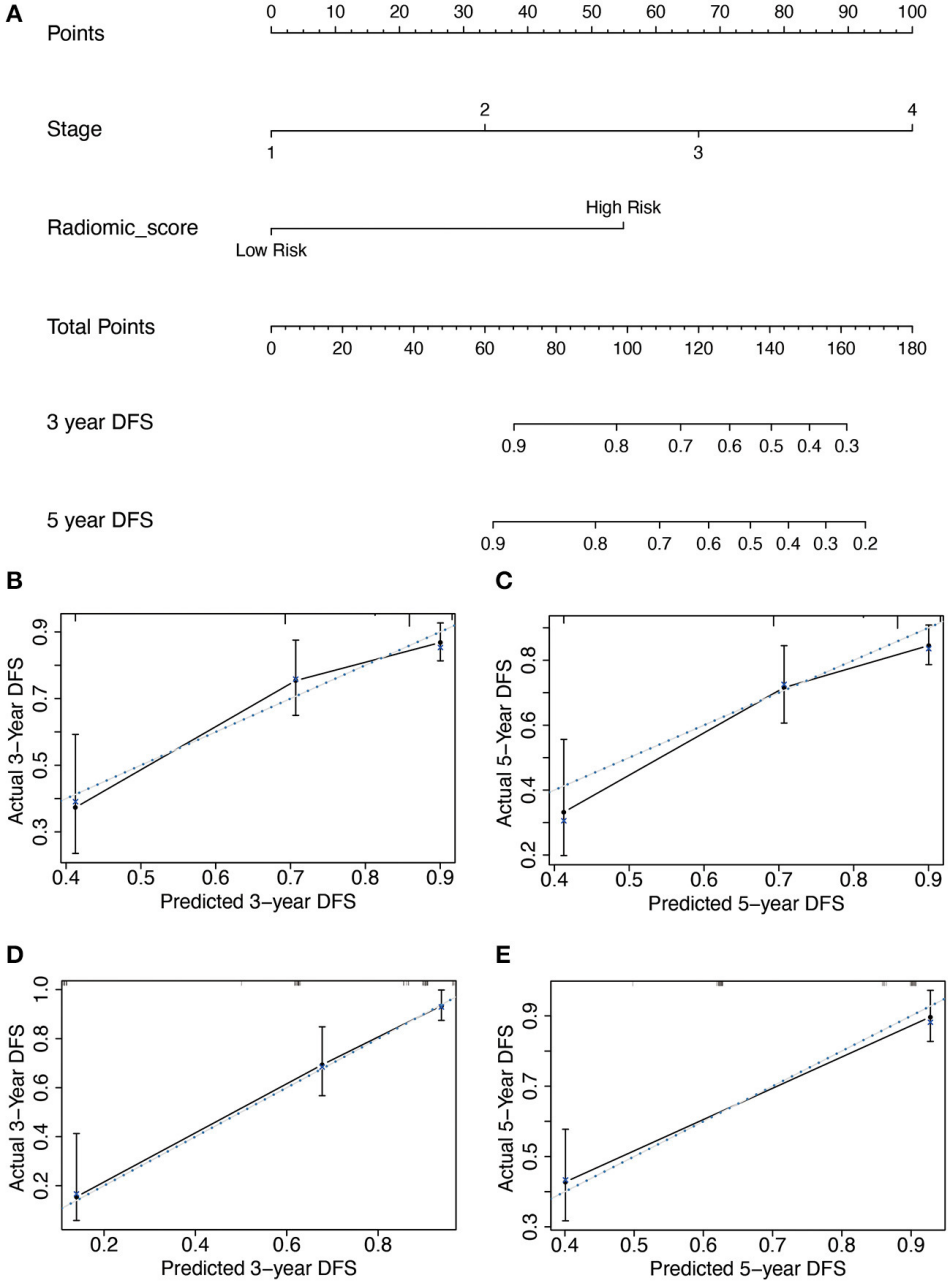
The developed nomogram incorporated Rad-score with TNM stage in the primary cohort. **(A)** The length of the coordinates for the prognostic factor was determined by the coefficient in the regression model. For every patient, the total score was calculated by summing every variable score. The probability of disease-free survival was derived from the mapping relationship between the evaluation results and total score on specified patient survival time. **(B–E)** Calibration curves of Rad-score based nomogram for 3 year DFS and 5 year DFS in the primary **(B,C)** and validation cohort **(D,E)**. The blue dot line is on the diagonal of the figure, indicating a complete fitting between the prognostic model and the actual data. The solid line illustrated the degree of fitting between model prediction and actual survival probability.

**Figure 4:**
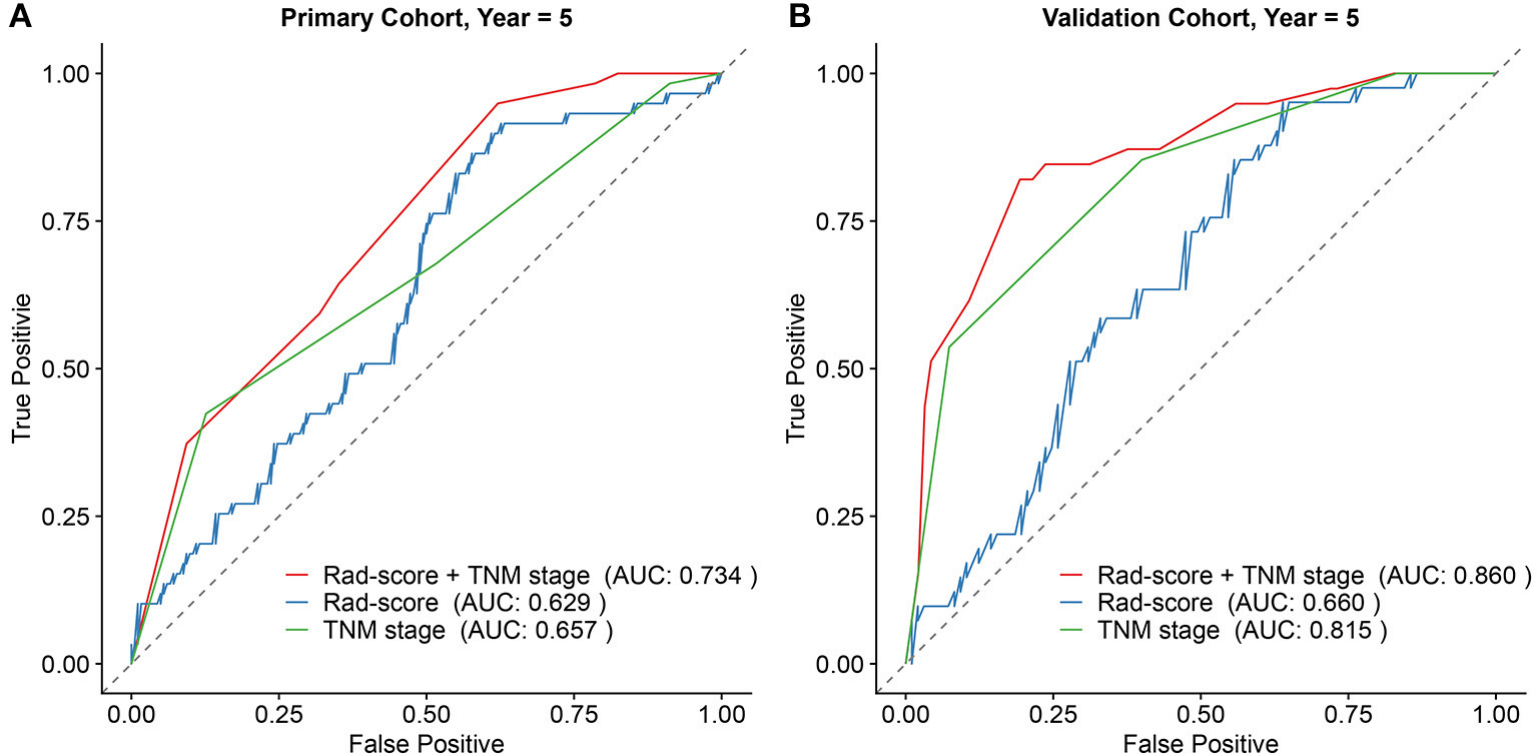
Comparison of the survival discriminative ability between Rad-score based nomogram and clinicopathological factor in the primary cohort **(A)** and validation cohort **(B)**.

Subsequently, based on the results of the multivariable analysis, a nomogram was developed combining the Rad-score and TNM stage (Figure 3A). To illustrate the performance of the nomogram prediction, calibration curves were used to evaluate the degree of fitting between the nomogram and the actual outcome of patients. The results showed that our nomogram showed good concordance between the predictive and actual survival probability in the primary (Figures 3B,C) and the validation cohort (Figures 3D,E). The C-index of the nomogram achieved 0.74 (0.69–0.80) in the primary cohort and 0.82 (0.77–0.87) in the validation cohort. To further confirm the effectiveness of the nomogram, we applied the receiver operating characteristic (ROC) to evaluate the discriminative ability of the nomogram for the 5 year DFS. The results showed that the area under curve (AUC) values of Rad-score incorporating the TNM stage reached 0.734 and 0.86 in the primary and validation cohort, respectively, outperforming the result of using the TNM stage alone (Figure 4).

### Radiomics Features Were Mainly Associated Metabolism-Related Pathway

**Figure 5:**
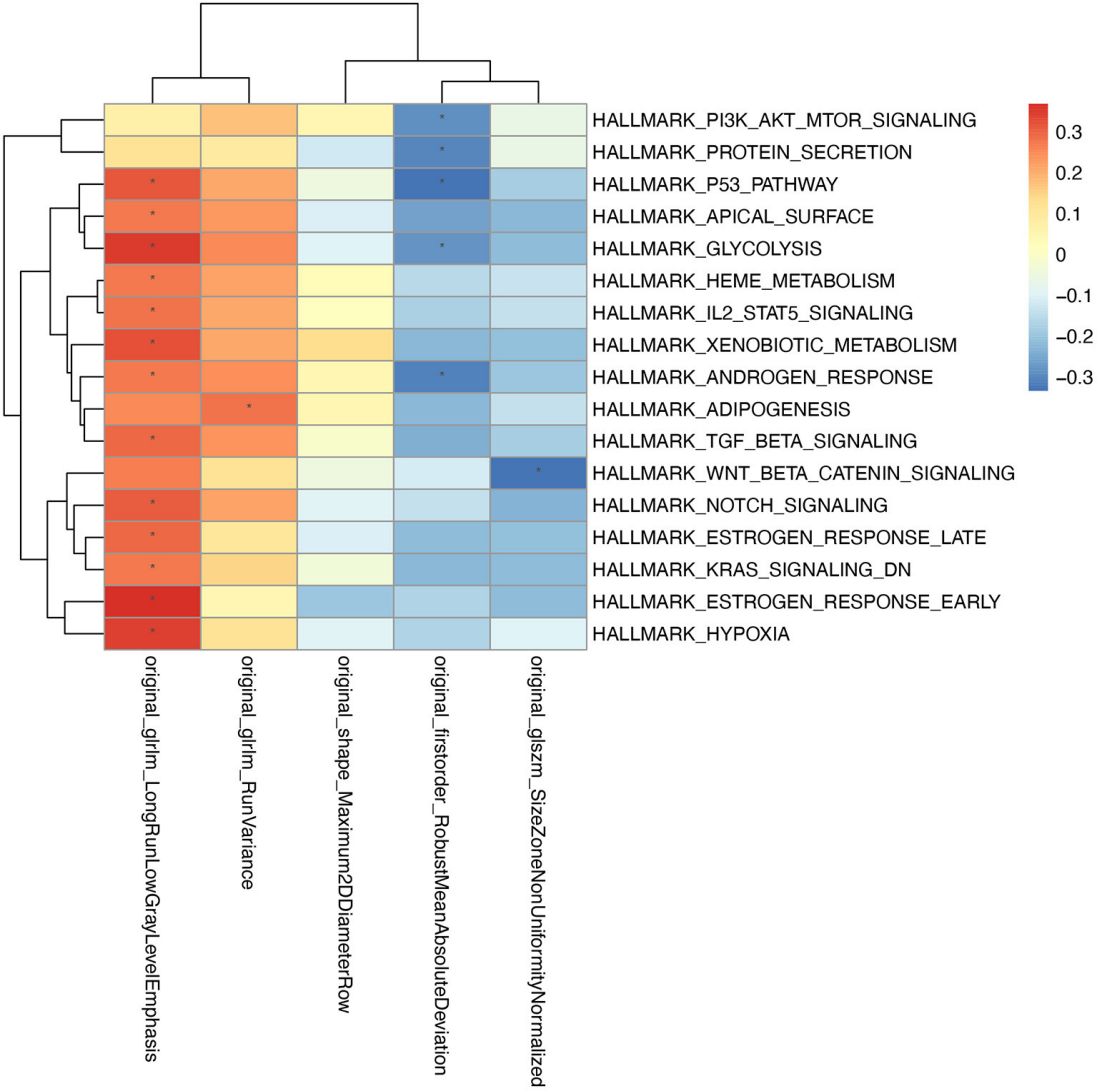
The association between radiomic features and gene expression profiles. For every patient, the enrichment scores of cancer related hallmarks were calculated by DeepCC package. The color block in the heatmap represent the value of Pearson's correlation coefficient between each hallmark and radiomic signature. The hallmarks significantly associated with radiomic features (*P* < 0.05) are marked with “*” in the heatmap.

To explore the association between radiomic features and the underlying biology mechanism, we performed the correlation analysis between the enrichment score of hallmarks and the 5 radiomics features. Gene expression data from 53 patients who have paired image data and RNA sequencing data were used to calculate the enrichment score of hallmarks by DeepCC. The pathways were selected according to the significant association with the radiomics signatures (Figure 5). Typically, the radiomics signatures showed significant enrichment in some metabolic pathways, such as protein secretion, glycolysis, heme metabolism, xenobiotic metabolism, adipogenesis.

## Discussion

Medical image analysis is a popular issue for precision therapy, which provides non-invasive information for clinical practice and treatment guidance. However, traditional medical image analysis can only find low throughput features or qualitative information manually by radiologists. Recent progress in machine learning enables researchers to extract high dimensional data quickly and quantitatively by radiomics. In this study, we used the radiomic features extracted from the CT image to predict the outcome of CRC patients. Survival analysis showed that high radiomics score was significantly associated with poor outcomes. Univariable and multivariable analyses confirmed the independent prognostic value of radiomic signature. Subsequently, the radiomics based nomogram was developed to predict the DFS, which showed better performance than using the TNM stage alone. Correlation analysis with gene expression profiles revealed that radiomic signature was mainly associated with metabolism-related pathways. Taken together, our results suggested that radiomic signature could be a supplement to the TNM stage for risk stratification of CRC patients.

Although the traditional gene expression-based molecular biomarkers have achieved good performance in many risks predicting tasks of colorectal cancer, there are still some difficulties that limit its clinical application (Walther et al., 2009; Kandimalla et al., 2018, 2019). Genetic test not only requires additional cost and time but also depend on the postoperative detection on pathological samples, which may limit the preoperative treatment intervention. These problems can be avoided by using medical image-based biomarkers. Recent progress in deep learning has generated a series of the image-based model with high accuracy and good performance (Kather et al., 2019; Lu et al., 2020; Skrede et al., 2020). However, a tricky problem of deep learning-based image model is the insufficiency of interpretation, which may raise concerns about its safety and limit its clinical application (Gordon et al., 2019). In contrast, radiomics is more interpretable and less dependent on sample size, which makes it easier to transform into clinical practice. Several studies have successfully use radiomics for individualized risk prediction of colorectal cancer (Liu et al., 2017, 2020). Furthermore, integration analysis of radiomics and gene expression profiles can provide deeper biological interpretation. Our results showed that the radiomics signatures showed significant enrichment in some metabolic pathways, which is an important mechanism for colorectal cancer initiation and progression (Satoh et al., 2017; Gao X. et al., 2019; Tang et al., 2019; La Vecchia and Sebastián, 2020). This indicated that the change of tumor metabolic status may cause morphological change on the image, which could be captured by radiomics features.

Our study not only established a robust radiomics-based nomogram for prognosis prediction of CRC, but also provided biological interpretation by correlation with gene expression profiles. However, there are still some limitations in our study. For example, the image and clinical data are collected from a single center, which may challenge the generalization of our model. Besides, as a retrospective study, the evidence level might be not enough. Prospective multicenter validation would be needed in future studies.

In conclusion, we proposed the Rad-score extracted from CT images as an independent prognostic factor for colorectal cancer. We incorporated Rad-score with the TNM stage to build a nomogram, which outperformed than TNM stage alone, indicating that the Rad-score can be complementary to the current staging strategies of CRC patients. As a non-invasive biomarker, our radiomics-based model can also provide a way of preoperative evaluation, which is helpful for clinical intervention.

## Data Availability Statement

The data supporting the findings of this study are available upon request from the corresponding authors (FG). The image data are not publicly available because they contain information that could compromise patient privacy. Gene expression data of COCC project are not publicly available currently and will be released by ICGC after milestone.

## Ethics Statement

The studies involving human participants were reviewed and approved by The Medical Ethics Committee of the Sixth Affiliated Hospital of Sun Yat-sen University. The ethics committee waived the requirement of written informed consent for participation.

## Author Contributions

FG and X-JW designed this study. DC, XD, and WW wrote the paper. DC analyzed and interpreted the data and drew the figures. XD extracted the radiomics features. WW, FG, and X-JW revised the paper. Z-PH, QZ, and M-EZ delineated the region of interest. M-YL, C-HL, and W-BK collected and cleaned up the clinical data. COCC Working Group provided assistance for data generation or analysis. All authors contributed to manuscript revision, read, and approved the submitted version.

## Conflict of Interest

The authors declare that the research was conducted in the absence of any commercial or financial relationships that could be construed as a potential conflict of interest.

## COCC Working Group

Xin-Juan Fan, Department of Pathology, The Sixth Affiliated Hospital of Sun Yat-sen University, Guangzhou, China; Guangdong Provincial Key Laboratory of Colorectal and Pelvic Floor Diseases, Supported by National Key Clinical Discipline, Guangdong Institute of Gastroenterology, Guangzhou, China

Xuan-Hui Liu, Department of Colorectal Surgery, The Sixth Affiliated Hospital of Sun Yat-sen University, Guangzhou, China; Guangdong Provincial Key Laboratory of Colorectal and Pelvic Floor Diseases, Supported by National Key Clinical Discipline, Guangdong Institute of Gastroenterology, Guangzhou, China

Jia Ke, Department of Colorectal Surgery, The Sixth Affiliated Hospital of Sun Yat-sen University, Guangzhou, China; Guangdong Provincial Key Laboratory of Colorectal and Pelvic Floor Diseases, Supported by National Key Clinical Discipline, Guangdong Institute of Gastroenterology, Guangzhou, China

Kui Wu, BGI-Shenzhen, Shenzhen, China

Lan-Xin Zhou, BGI-Shenzhen, Shenzhen, China

Shu-Zhen Luo, BGI-Shenzhen, Shenzhen, China

Dong-Bing Liu, BGI-Shenzhen, Shenzhen, China

Xin Wang, Department of Biomedical Sciences, City University of Hong Kong, Hong Kong, China

Tan Wu, Department of Biomedical Sciences, City University of Hong Kong, Hong Kong, China

Zhong-Xu Zhu, Department of Biomedical Sciences, City University of Hong Kong, Hong Kong, China

Rong-Hui He, Hangzhou YITU Healthcare Technology Co., Ltd, Hangzhou, China

Lan-Xuan Liu, Hangzhou YITU Healthcare Technology Co., Ltd, Hangzhou, China

Ming Li, Hangzhou YITU Healthcare Technology Co., Ltd, Hangzhou, China

## References

[B1] Aerts H. J. Velazquez E. R. Leijenaar R. T. Parmar C. Grossmann P. Carvalho S.. (2014). Decoding tumour phenotype by noninvasive imaging using a quantitative radiomics approach. Nat. Commun. 5:4006. 10.1038/ncomms5006 24892406 PMC4059926

[B2] Farhidzadeh H. Kim J. Y. Scott J. G. Goldgof D. B. Hall L. O. Harrison L. B. (Eds.). (2016). “Classification of progression free survival with nasopharyngeal carcinoma tumors,” in Medical Imaging 2016: Computer-Aided Diagnosis (San Diego, CA: International Society for Optics and Photonics).

[B3] Friedman J. Hastie T. Tibshirani R. (2010). Regularization paths for generalized linear models via coordinate descent. J. Stat. Softw. 33, 1–22. 10.18637/jss.v033.i01 20808728 PMC2929880

[B4] Gao F. Wang W. Tan M. Zhu L. Zhang Y. Fessler E.. (2019). DeepCC: a novel deep learning-based framework for cancer molecular subtype classification. Oncogenesis 8:44. 10.1038/s41389-019-0157-8 31420533 PMC6697729

[B5] Gao X. Sanderson S. M. Dai Z. Reid M. A. Cooper D. E. Lu M.. (2019). Dietary methionine influences therapy in mouse cancer models and alters human metabolism. Nature 572, 397–401. 10.1038/s41586-019-1437-3 31367041 PMC6951023

[B6] Gordon L. Grantcharov T. Rudzicz F. (2019). Explainable artificial intelligence for safe intraoperative decision support. JAMA Surg. 154, 1064–1065. 10.1001/jamasurg.2019.2821 31509185

[B7] Harrell F. E. Jr. (2016). rms: Regression Modeling Strategies. R package version, 5.

[B8] Heagerty P. J. Lumley T. Pepe M. S. (2000). Time-dependent ROC curves for censored survival data and a diagnostic marker. Biometrics 56, 337–344. 10.1111/j.0006-341X.2000.00337.x 10877287

[B9] Huang Y. Liu Z. He L. Chen X. Pan D. Ma Z.. (2016). Radiomics signature: a potential biomarker for the prediction of disease-free survival in early-stage (I or II) non-small cell lung cancer. Radiology 281, 947–957. 10.1148/radiol.2016152234 27347764

[B10] Huang Y. Q. Liang C. H. He L. Tian J. Liang C. S. Chen X.. (2016). Development and validation of a radiomics nomogram for preoperative prediction of lymph node metastasis in colorectal cancer. J. Clin. Oncol. 34, 2157–2164. 10.1200/JCO.2015.65.9128 27138577

[B11] Kandimalla R. Gao F. Matsuyama T. Ishikawa T. Uetake H. Takahashi N.. (2018). Genome-wide discovery and identification of a novel miRNA signature for recurrence prediction in stage II and III colorectal cancer. Clin. Cancer Res. 24, 3867–3877. 10.1158/1078-0432.CCR-17-3236 29514841 PMC6095767

[B12] Kandimalla R. Ozawa T. Gao F. Wang X. Goel A. (2019). Gene expression signature in surgical tissues and endoscopic biopsies identifies high-risk T1 colorectal cancers. Gastroenterology 156, 2338–2341.e3. 10.1053/j.gastro.2019.02.027 30797795 PMC6538250

[B13] Kather J. N. Krisam J. Charoentong P. Luedde T. Herpel E. Weis C. A.. (2019). Predicting survival from colorectal cancer histology slides using deep learning: a retrospective multicenter study. PLoS Med. 16:e1002730. 10.1371/journal.pmed.1002730 30677016 PMC6345440

[B14] Kim J. E. Lee J. M. Baek J. H. Moon S. K. Kim S. H. Han J. K. (2015). Differentiation of poorly differentiated colorectal adenocarcinomas from well- or moderately differentiated colorectal adenocarcinomas at contrast-enhanced multidetector CT. Abdom. Imaging 40, 1–10. 10.1007/s00261-014-0176-z 24990513

[B15] Kumar V. Gu Y. Basu S. Berglund A. Eschrich S. A. Schabath M. B.. (2012). Radiomics: the process and the challenges. Magn. Reson. Imaging 30, 1234–1248. 10.1016/j.mri.2012.06.010 22898692 PMC3563280

[B16] La Vecchia S. Sebastián C. (2020). Metabolic pathways regulating colorectal cancer initiation and progression. Semin. Cell Dev. Biol. 98, 63–70. 10.1016/j.semcdb.2019.05.018 31129171

[B17] Lambin P. Leijenaar R. T. H. Deist T. M. Peerlings J. de Jong E. E. C. van Timmeren J.. (2017). Radiomics: the bridge between medical imaging and personalized medicine. Nat. Rev. Clin. Oncol. 14, 749–762. 10.1038/nrclinonc.2017.141 28975929

[B18] Liang C. Huang Y. He L. Chen X. Ma Z. Dong D.. (2016). The development and validation of a CT-based radiomics signature for the preoperative discrimination of stage I-II and stage III-IV colorectal cancer. Oncotarget 7, 31401–31412. 10.18632/oncotarget.8919 27120787 PMC5058766

[B19] Limkin E. J. Sun R. Dercle L. Zacharaki E. I. Robert C. Reuz,é S.. (2017). Promises and challenges for the implementation of computational medical imaging (radiomics) in oncology. Ann. Oncol. 28, 1191–1206. 10.1093/annonc/mdx034 28168275

[B20] Liu Z. Meng X. Zhang H. Li Z. Liu J. Sun K.. (2020). Predicting distant metastasis and chemotherapy benefit in locally advanced rectal cancer. Nat. Commun. 11:4308. 10.1038/s41467-020-18162-9 32855399 PMC7452897

[B21] Liu Z. Zhang X. Y. Shi Y. J. Wang L. Zhu H. T. Tang Z.. (2017). Radiomics analysis for evaluation of pathological complete response to neoadjuvant chemoradiotherapy in locally advanced rectal cancer. Clin. Cancer Res. 23, 7253–7262. 10.1158/1078-0432.CCR-17-1038 28939744

[B22] Lu M. T. Raghu V. K. Mayrhofer T. Aerts H. Hoffmann U. (2020). Deep learning using chest radiographs to identify high-risk smokers for lung cancer screening computed tomography: development and validation of a prediction model. Ann. Intern. Med. 173, 704–713. 10.7326/M20-1868 32866413 PMC9200444

[B23] Moghimi-Dehkordi B. Safaee A. (2012). An overview of colorectal cancer survival rates and prognosis in Asia. World J. Gastrointest. Oncol. 4, 71–75. 10.4251/wjgo.v4.i4.71 22532879 PMC3334382

[B24] Satoh K. Yachida S. Sugimoto M. Oshima M. Nakagawa T. Akamoto S.. (2017). Global metabolic reprogramming of colorectal cancer occurs at adenoma stage and is induced by MYC. Proc. Natl. Acad. Sci. U.S.A. 114, E7697–E7706. 10.1073/pnas.1710366114 28847964 PMC5604037

[B25] Segal E. Sirlin C. B. Ooi C. Adler A. S. Gollub J. Chen X.. (2007). Decoding global gene expression programs in liver cancer by noninvasive imaging. Nat. Biotechnol. 25, 675–680. 10.1038/nbt1306 17515910

[B26] Siegel R. L. Miller K. D. Goding Sauer A. Fedewa S. A. Butterly L. F. Anderson J. C. (2020). Colorectal cancer statistics, 2020. CA Cancer J. Clin. 70, 145–164. 10.3322/caac.21601 32133645

[B27] Skrede O. J. De Raedt S. Kleppe A. Hveem T. S. Liestøl K. Maddison J.. (2020). Deep learning for prediction of colorectal cancer outcome: a discovery and validation study. Lancet 395, 350–360. 10.1016/S0140-6736(19)32998-8 32007170

[B28] Tang J. Yan T. Bao Y. Shen C. Yu C. Zhu X.. (2019). LncRNA GLCC1 promotes colorectal carcinogenesis and glucose metabolism by stabilizing c-Myc. Nat. Commun. 10:3499. 10.1038/s41467-019-11447-8 31375671 PMC6677832

[B29] van Griethuysen J. J. M. Fedorov A. Parmar C. Hosny A. Aucoin N. Narayan V.. (2017). Computational radiomics system to decode the radiographic phenotype. Cancer Res. 77, e104–e107. 10.1158/0008-5472.CAN-17-0339 29092951 PMC5672828

[B30] Walther A. Johnstone E. Swanton C. Midgley R. Tomlinson I. Kerr D. (2009). Genetic prognostic and predictive markers in colorectal cancer. Nat. Rev. Cancer 9, 489–499. 10.1038/nrc2645 19536109

