# Supplementary material for: A Metabolism-Related Radiomics Signature for Predicting the Prognosis of Colorectal Cancer
Source: Front Mol Biosci. 2021 Jan 7;7:613918. doi: 10.3389/fmolb.2020.613918 (PMC7817969; doi:10.3389/fmolb.2020.613918)
Supplement: Supplementary file 1 [file Table_1.DOCX]

Supplemental Table 1 The groups and Rad-score of patients in this study.

| **ID** | **Cohort** | **Risk Group** | **Rad-score** | **With gene expression data** |
| --- | --- | --- | --- | --- |
| **1** | Primary | Low Risk | -0.142 | No |
| **2** | Primary | High Risk | -0.032 | No |
| **3** | Primary | Low Risk | -0.089 | No |
| **4** | Primary | Low Risk | -0.126 | No |
| **5** | Primary | High Risk | 0.049 | No |
| **6** | Primary | High Risk | -0.041 | No |
| **7** | Primary | Low Risk | -0.078 | No |
| **8** | Primary | Low Risk | -0.116 | No |
| **9** | Primary | High Risk | 0.004 | No |
| **10** | Primary | High Risk | 0.132 | No |
| **11** | Primary | High Risk | -0.044 | No |
| **12** | Primary | High Risk | -0.024 | No |
| **13** | Primary | High Risk | -0.057 | No |
| **14** | Primary | High Risk | -0.037 | No |
| **15** | Primary | High Risk | -0.07 | No |
| **16** | Primary | Low Risk | -0.298 | No |
| **17** | Primary | Low Risk | -0.266 | No |
| **18** | Primary | High Risk | 0.093 | No |
| **19** | Primary | High Risk | -0.073 | No |
| **20** | Primary | High Risk | 0.088 | No |
| **21** | Primary | High Risk | 0.371 | No |
| **22** | Primary | High Risk | 0.02 | No |
| **23** | Primary | High Risk | 0.231 | No |
| **24** | Primary | High Risk | 0.188 | No |
| **25** | Primary | Low Risk | -0.2 | No |
| **26** | Primary | High Risk | 0.127 | No |
| **27** | Primary | High Risk | -0.021 | No |
| **28** | Primary | High Risk | -0.071 | No |
| **29** | Primary | Low Risk | -0.208 | No |
| **30** | Primary | High Risk | 0.214 | No |
| **31** | Primary | High Risk | 0.009 | No |
| **32** | Primary | High Risk | 1.041 | No |
| **33** | Primary | High Risk | 0.058 | No |
| **34** | Primary | Low Risk | -0.101 | No |
| **35** | Primary | High Risk | 0.13 | No |
| **36** | Primary | Low Risk | -0.109 | No |
| **37** | Primary | High Risk | 0.323 | No |
| **38** | Primary | Low Risk | -0.203 | No |
| **39** | Primary | High Risk | 0.083 | No |
| **40** | Primary | High Risk | -0.023 | No |
| **41** | Primary | High Risk | -0.012 | No |
| **42** | Primary | Low Risk | -0.084 | No |
| **43** | Primary | Low Risk | -0.123 | No |
| **44** | Primary | Low Risk | -0.155 | No |
| **45** | Primary | High Risk | -0.056 | No |
| **46** | Primary | High Risk | -0.016 | No |
| **47** | Primary | Low Risk | -0.091 | No |
| **48** | Primary | High Risk | -0.005 | No |
| **49** | Primary | Low Risk | -0.132 | No |
| **50** | Primary | High Risk | -0.059 | No |
| **51** | Primary | High Risk | -0.035 | No |
| **52** | Primary | Low Risk | -0.383 | No |
| **53** | Primary | Low Risk | -0.255 | No |
| **54** | Primary | High Risk | 0.075 | No |
| **55** | Primary | High Risk | -0.017 | No |
| **56** | Primary | High Risk | 0.055 | No |
| **57** | Primary | High Risk | -0.037 | No |
| **58** | Primary | High Risk | -0.07 | No |
| **59** | Primary | High Risk | 0.089 | No |
| **60** | Primary | Low Risk | -0.189 | No |
| **61** | Primary | High Risk | 0.251 | No |
| **62** | Primary | High Risk | 0.1 | No |
| **63** | Primary | High Risk | 0.068 | No |
| **64** | Primary | High Risk | 0.267 | No |
| **65** | Primary | High Risk | 0.034 | No |
| **66** | Primary | High Risk | -0.055 | No |
| **67** | Primary | High Risk | 0.083 | No |
| **68** | Primary | High Risk | 0.145 | No |
| **69** | Primary | High Risk | -0.02 | No |
| **70** | Primary | Low Risk | -0.183 | No |
| **71** | Primary | High Risk | 0.115 | No |
| **72** | Primary | Low Risk | -0.118 | No |
| **73** | Primary | High Risk | -0.034 | No |
| **74** | Primary | High Risk | 0.141 | No |
| **75** | Primary | Low Risk | -0.174 | No |
| **76** | Primary | High Risk | 0.182 | No |
| **77** | Primary | High Risk | 0.071 | No |
| **78** | Primary | High Risk | 0.048 | No |
| **79** | Primary | High Risk | 0.001 | No |
| **80** | Primary | Low Risk | -0.118 | No |
| **81** | Primary | Low Risk | -0.16 | No |
| **82** | Primary | High Risk | 0.024 | No |
| **83** | Primary | High Risk | -0.048 | No |
| **84** | Primary | High Risk | 0.174 | No |
| **85** | Primary | Low Risk | -0.208 | No |
| **86** | Primary | Low Risk | -0.236 | No |
| **87** | Primary | Low Risk | -0.167 | No |
| **88** | Primary | High Risk | 0.214 | No |
| **89** | Primary | Low Risk | -0.164 | No |
| **90** | Primary | High Risk | 0.091 | No |
| **91** | Primary | Low Risk | -0.094 | No |
| **92** | Primary | High Risk | -0.007 | No |
| **93** | Primary | High Risk | -0.067 | No |
| **94** | Primary | High Risk | -0.033 | No |
| **95** | Primary | High Risk | -0.031 | No |
| **96** | Primary | High Risk | 0.154 | No |
| **97** | Primary | Low Risk | -0.107 | No |
| **98** | Primary | High Risk | 0.124 | No |
| **99** | Primary | High Risk | -0.032 | No |
| **100** | Primary | Low Risk | -0.113 | No |
| **101** | Primary | High Risk | -0.04 | No |
| **102** | Primary | Low Risk | -0.118 | No |
| **103** | Primary | High Risk | -0.006 | No |
| **104** | Primary | High Risk | 0.102 | No |
| **105** | Primary | High Risk | 0.128 | No |
| **106** | Primary | High Risk | 0.142 | No |
| **107** | Primary | High Risk | 0.141 | No |
| **108** | Primary | High Risk | 0.07 | No |
| **109** | Primary | High Risk | 0.127 | No |
| **110** | Primary | Low Risk | -0.149 | No |
| **111** | Primary | High Risk | 0.153 | No |
| **112** | Primary | High Risk | 0.156 | No |
| **113** | Primary | High Risk | 0.166 | No |
| **114** | Primary | High Risk | 0.149 | No |
| **115** | Primary | High Risk | -0.06 | No |
| **116** | Primary | High Risk | -0.018 | No |
| **117** | Primary | High Risk | -0.057 | No |
| **118** | Primary | High Risk | 0.014 | No |
| **119** | Primary | Low Risk | -0.152 | No |
| **120** | Primary | High Risk | 0.004 | No |
| **121** | Primary | High Risk | 0.063 | No |
| **122** | Primary | High Risk | 0.045 | No |
| **123** | Primary | High Risk | 0.022 | No |
| **124** | Primary | Low Risk | -0.097 | No |
| **125** | Primary | High Risk | -0.042 | No |
| **126** | Primary | High Risk | 0.155 | No |
| **127** | Primary | High Risk | -0.026 | No |
| **128** | Primary | High Risk | 0.091 | No |
| **129** | Primary | High Risk | -0.014 | No |
| **130** | Primary | High Risk | 0.11 | No |
| **131** | Primary | High Risk | 0.238 | No |
| **132** | Primary | High Risk | 0.107 | No |
| **133** | Primary | High Risk | 0.11 | No |
| **134** | Primary | High Risk | 0.052 | No |
| **135** | Primary | Low Risk | -0.093 | No |
| **136** | Primary | High Risk | 0.236 | No |
| **137** | Primary | High Risk | -0.002 | No |
| **138** | Primary | High Risk | 0.198 | No |
| **139** | Primary | High Risk | -0.009 | No |
| **140** | Primary | High Risk | 0.061 | No |
| **141** | Primary | High Risk | 0.267 | No |
| **142** | Primary | High Risk | 0.081 | No |
| **143** | Primary | High Risk | 0.103 | No |
| **144** | Primary | High Risk | 0.114 | No |
| **145** | Primary | Low Risk | -0.24 | No |
| **146** | Primary | Low Risk | -0.077 | No |
| **147** | Primary | High Risk | 0.08 | No |
| **148** | Primary | High Risk | 0.026 | No |
| **149** | Primary | Low Risk | -0.136 | No |
| **150** | Primary | High Risk | 0.098 | No |
| **151** | Primary | High Risk | -0.007 | No |
| **152** | Primary | High Risk | 0.01 | No |
| **153** | Primary | High Risk | 0.07 | No |
| **154** | Primary | High Risk | 0.316 | No |
| **155** | Primary | High Risk | 0.092 | No |
| **156** | Primary | High Risk | 0.033 | No |
| **157** | Primary | High Risk | 0.086 | No |
| **158** | Primary | High Risk | 0.178 | No |
| **159** | Primary | Low Risk | -0.104 | No |
| **160** | Primary | High Risk | 0.156 | No |
| **161** | Primary | High Risk | 0.928 | No |
| **162** | Primary | High Risk | -0.037 | No |
| **163** | Primary | High Risk | -0.045 | No |
| **164** | Primary | High Risk | 0.035 | No |
| **165** | Primary | High Risk | 0.033 | No |
| **166** | Primary | High Risk | 0.032 | No |
| **167** | Primary | High Risk | 0.033 | No |
| **168** | Primary | High Risk | 0.118 | No |
| **169** | Primary | High Risk | 0.145 | No |
| **170** | Primary | High Risk | 0.003 | No |
| **171** | Primary | High Risk | 0.03 | No |
| **172** | Primary | High Risk | 0.084 | No |
| **173** | Primary | High Risk | -0.023 | No |
| **174** | Primary | High Risk | 0.035 | No |
| **175** | Primary | Low Risk | -0.108 | No |
| **176** | Primary | High Risk | 0.019 | No |
| **177** | Primary | High Risk | 0.215 | No |
| **178** | Primary | High Risk | 0.007 | No |
| **179** | Primary | High Risk | 0.296 | No |
| **180** | Primary | High Risk | 0.223 | No |
| **181** | Primary | High Risk | 0.063 | No |
| **182** | Primary | High Risk | -0.023 | No |
| **183** | Primary | High Risk | 0.116 | No |
| **184** | Primary | High Risk | 0.059 | No |
| **185** | Primary | High Risk | 0.011 | No |
| **186** | Primary | High Risk | -0.035 | No |
| **187** | Primary | High Risk | 0.025 | No |
| **188** | Primary | High Risk | -0.062 | No |
| **189** | Primary | High Risk | -0.031 | No |
| **190** | Primary | High Risk | 0.218 | No |
| **191** | Primary | High Risk | 0.399 | No |
| **192** | Primary | High Risk | 0.151 | No |
| **193** | Primary | High Risk | 0.016 | No |
| **194** | Primary | Low Risk | -0.157 | No |
| **195** | Primary | High Risk | -0.043 | No |
| **196** | Primary | Low Risk | -0.159 | No |
| **197** | Primary | Low Risk | -0.08 | No |
| **198** | Primary | Low Risk | -0.201 | No |
| **199** | Primary | High Risk | -0.04 | No |
| **200** | Primary | Low Risk | -0.124 | No |
| **201** | Primary | Low Risk | -0.259 | No |
| **202** | Primary | Low Risk | -0.147 | No |
| **203** | Primary | Low Risk | -0.187 | No |
| **204** | Primary | Low Risk | -0.168 | No |
| **205** | Primary | High Risk | -0.063 | No |
| **206** | Primary | Low Risk | -0.304 | No |
| **207** | Primary | Low Risk | -0.077 | No |
| **208** | Primary | Low Risk | -0.082 | No |
| **209** | Primary | Low Risk | -0.711 | No |
| **210** | Primary | Low Risk | -0.223 | No |
| **211** | Primary | Low Risk | -0.195 | No |
| **212** | Primary | Low Risk | -0.142 | No |
| **213** | Primary | Low Risk | -0.19 | No |
| **214** | Primary | Low Risk | -0.153 | No |
| **215** | Primary | Low Risk | -0.124 | No |
| **216** | Primary | Low Risk | -0.197 | No |
| **217** | Primary | Low Risk | -0.324 | No |
| **218** | Primary | High Risk | 0.046 | No |
| **219** | Primary | High Risk | -0.077 | No |
| **220** | Primary | High Risk | -0.077 | No |
| **221** | Primary | Low Risk | -0.082 | No |
| **222** | Primary | Low Risk | -0.123 | No |
| **223** | Primary | Low Risk | -0.106 | No |
| **224** | Primary | Low Risk | -0.091 | No |
| **225** | Primary | High Risk | 0.127 | No |
| **226** | Primary | High Risk | -0.037 | No |
| **227** | Primary | High Risk | 0.076 | No |
| **228** | Primary | High Risk | 0.033 | No |
| **229** | Primary | High Risk | 0.23 | No |
| **230** | Primary | Low Risk | -0.351 | No |
| **231** | Primary | High Risk | 0.048 | No |
| **232** | Primary | Low Risk | -0.12 | No |
| **233** | Primary | High Risk | 0.052 | No |
| **234** | Primary | Low Risk | -0.252 | No |
| **235** | Primary | High Risk | 0.051 | No |
| **236** | Primary | High Risk | 0.105 | No |
| **237** | Primary | High Risk | 0.042 | No |
| **238** | Primary | Low Risk | -0.165 | No |
| **239** | Primary | High Risk | -0.064 | No |
| **240** | Primary | High Risk | -0.028 | No |
| **241** | Primary | Low Risk | -0.103 | No |
| **242** | Primary | Low Risk | -0.168 | No |
| **243** | Validation | High Risk | 0.134 | Yes |
| **244** | Validation | High Risk | 0.017 | Yes |
| **245** | Validation | High Risk | 0.036 | Yes |
| **246** | Validation | High Risk | 0.061 | No |
| **247** | Validation | High Risk | 0.045 | Yes |
| **248** | Validation | High Risk | 0.059 | Yes |
| **249** | Validation | High Risk | 0.102 | Yes |
| **250** | Validation | High Risk | 0.361 | Yes |
| **251** | Validation | High Risk | -0.026 | Yes |
| **252** | Validation | Low Risk | -0.162 | Yes |
| **253** | Validation | High Risk | 0.021 | Yes |
| **254** | Validation | High Risk | -0.049 | Yes |
| **255** | Validation | High Risk | -0.013 | Yes |
| **256** | Validation | High Risk | 0.147 | Yes |
| **257** | Validation | High Risk | 0.078 | Yes |
| **258** | Validation | High Risk | -0.065 | Yes |
| **259** | Validation | Low Risk | -0.235 | Yes |
| **260** | Validation | Low Risk | -0.141 | Yes |
| **261** | Validation | High Risk | 0.11 | Yes |
| **262** | Validation | High Risk | -0.017 | Yes |
| **263** | Validation | High Risk | 0.12 | Yes |
| **264** | Validation | High Risk | 0.119 | Yes |
| **265** | Validation | Low Risk | -0.112 | Yes |
| **266** | Validation | High Risk | -0.073 | Yes |
| **267** | Validation | High Risk | 0.014 | Yes |
| **268** | Validation | High Risk | 0.018 | Yes |
| **269** | Validation | High Risk | 0.055 | Yes |
| **270** | Validation | High Risk | -0.049 | Yes |
| **271** | Validation | High Risk | 0.098 | Yes |
| **272** | Validation | High Risk | 0.03 | Yes |
| **273** | Validation | High Risk | 0.148 | Yes |
| **274** | Validation | High Risk | 0.024 | Yes |
| **275** | Validation | High Risk | 0.241 | No |
| **276** | Validation | High Risk | -0.02 | Yes |
| **277** | Validation | High Risk | 0.101 | Yes |
| **278** | Validation | High Risk | -0.012 | Yes |
| **279** | Validation | High Risk | -0.003 | Yes |
| **280** | Validation | High Risk | 0.383 | Yes |
| **281** | Validation | High Risk | -0.056 | Yes |
| **282** | Validation | High Risk | 0.169 | Yes |
| **283** | Validation | Low Risk | -0.167 | Yes |
| **284** | Validation | High Risk | 0.04 | Yes |
| **285** | Validation | High Risk | -0.032 | Yes |
| **286** | Validation | High Risk | 0.157 | Yes |
| **287** | Validation | High Risk | 0 | Yes |
| **288** | Validation | High Risk | -0.023 | Yes |
| **289** | Validation | High Risk | 0.036 | Yes |
| **290** | Validation | High Risk | 0.12 | No |
| **291** | Validation | Low Risk | -0.102 | No |
| **292** | Validation | High Risk | -0.048 | No |
| **293** | Validation | High Risk | -0.048 | No |
| **294** | Validation | Low Risk | -0.242 | No |
| **295** | Validation | High Risk | -0.025 | No |
| **296** | Validation | High Risk | 0.044 | No |
| **297** | Validation | High Risk | -0.031 | No |
| **298** | Validation | High Risk | 0.004 | No |
| **299** | Validation | High Risk | 0.088 | No |
| **300** | Validation | High Risk | 0.032 | No |
| **301** | Validation | High Risk | -0.05 | No |
| **302** | Validation | High Risk | -0.03 | No |
| **303** | Validation | High Risk | 0.022 | No |
| **304** | Validation | High Risk | 0.235 | No |
| **305** | Validation | Low Risk | -0.104 | No |
| **306** | Validation | High Risk | 0.162 | No |
| **307** | Validation | Low Risk | -0.098 | No |
| **308** | Validation | High Risk | -0.032 | No |
| **309** | Validation | High Risk | 0.008 | No |
| **310** | Validation | High Risk | 0.126 | No |
| **311** | Validation | Low Risk | -0.213 | No |
| **312** | Validation | High Risk | 0.107 | No |
| **313** | Validation | Low Risk | -0.139 | No |
| **314** | Validation | High Risk | 0.013 | No |
| **315** | Validation | Low Risk | -0.322 | No |
| **316** | Validation | High Risk | 0.233 | No |
| **317** | Validation | High Risk | 0.029 | No |
| **318** | Validation | Low Risk | -0.195 | No |
| **319** | Validation | High Risk | 0.021 | No |
| **320** | Validation | Low Risk | -0.156 | No |
| **321** | Validation | High Risk | 0.016 | No |
| **322** | Validation | High Risk | -0.061 | No |
| **323** | Validation | Low Risk | -0.157 | No |
| **324** | Validation | High Risk | 0.129 | No |
| **325** | Validation | High Risk | -0.03 | No |
| **326** | Validation | High Risk | -0.066 | No |
| **327** | Validation | High Risk | 0.084 | No |
| **328** | Validation | High Risk | 0.186 | No |
| **329** | Validation | High Risk | 0.027 | No |
| **330** | Validation | Low Risk | -0.095 | No |
| **331** | Validation | High Risk | 0.259 | No |
| **332** | Validation | High Risk | -0.051 | No |
| **333** | Validation | High Risk | 0.061 | No |
| **334** | Validation | High Risk | 0.007 | No |
| **335** | Validation | High Risk | -0.054 | No |
| **336** | Validation | High Risk | 0.105 | No |
| **337** | Validation | High Risk | 0.184 | No |
| **338** | Validation | Low Risk | -0.081 | No |
| **339** | Validation | Low Risk | -0.122 | No |
| **340** | Validation | High Risk | 0.025 | No |
| **341** | Validation | High Risk | -0.011 | No |
| **342** | Validation | High Risk | -0.076 | No |
| **343** | Validation | High Risk | -0.077 | No |
| **344** | Validation | Low Risk | -0.136 | No |
| **345** | Validation | Low Risk | -0.141 | No |
| **346** | Validation | High Risk | -0.039 | No |
| **347** | Validation | Low Risk | -0.233 | No |
| **348** | Validation | High Risk | -0.059 | No |
| **349** | Validation | Low Risk | -0.149 | No |
| **350** | Validation | Low Risk | -0.142 | No |
| **351** | Validation | Low Risk | -0.145 | No |
| **352** | Validation | High Risk | 0.008 | No |
| **353** | Validation | High Risk | 0.166 | No |
| **354** | Validation | Low Risk | -0.087 | No |
| **355** | Validation | High Risk | -0.057 | No |
| **356** | Validation | Low Risk | -0.108 | No |
| **357** | Validation | High Risk | 0.088 | No |
| **358** | Validation | High Risk | 0.591 | No |
| **359** | Validation | High Risk | -0.062 | No |
| **360** | Validation | High Risk | 0.067 | No |
| **361** | Validation | High Risk | 0.181 | No |
| **362** | Validation | High Risk | 0.055 | No |
| **363** | Validation | High Risk | -0.026 | No |
| **364** | Validation | Low Risk | -0.147 | No |
| **365** | Validation | Low Risk | -0.114 | No |
| **366** | Validation | Low Risk | -0.086 | No |
| **367** | Validation | Low Risk | -0.093 | No |
| **368** | Validation | Low Risk | -0.186 | Yes |
| **369** | Validation | High Risk | 0.111 | Yes |
| **370** | Validation | High Risk | -0.044 | Yes |
| **371** | Validation | Low Risk | -0.22 | No |
| **372** | Validation | High Risk | -0.01 | Yes |
| **373** | Validation | High Risk | 0.038 | No |
| **374** | Validation | Low Risk | -0.171 | No |
| **375** | Validation | Low Risk | -0.145 | No |
| **376** | Validation | High Risk | 0.045 | No |
| **377** | Validation | Low Risk | -0.307 | Yes |
| **378** | Validation | High Risk | -0.006 | Yes |
| **379** | Validation | High Risk | 0.253 | No |
| **380** | Validation | High Risk | -0.069 | Yes |
| **381** | Validation | High Risk | 0.106 | Yes |
